# Tumor Vaccines for Malignant Melanoma: Progress, Challenges, and Future Directions

**DOI:** 10.32604/or.2025.063843

**Published:** 2025-07-18

**Authors:** Wenfei Luo, Dingming Song, Yibo He, Judong Song, Yunzhen Ding

**Affiliations:** 1 Department of Dermatology, Jinzhou Medical University, The First Affiliated Hospital of Jinzhou Medical University, Jinzhou, 121001, China; 2 Department of Urology, Jinzhou Medical University, The First Affiliated Hospital of Jinzhou Medical University, Jinzhou, 121001, China; 3 Department of Clinical Lab, The First Affiliated Hospital of Zhejiang Chinese Medical University (Zhejiang Provincial Hospital of Chinese Medicine), Hangzhou, 310060, China; 4 Department of Pharmacy, The First People’s Hospital of Lin’an District, Hangzhou, Lin’an People’s Hospital Affiliated to Hangzhou Medical College, Hangzhou, 311399, China; 5 Department of Postpartum Rehabilitation, The First People’s Hospital of Lin’an District, Hangzhou, Lin’an People’s Hospital Affiliated to Hangzhou Medical College, Hangzhou, 311399, China

**Keywords:** Malignant melanoma, tumor vaccines, peptide vaccines, DNA/RNA vaccines, dendritic cell vaccines, tumor cell vaccines

## Abstract

Malignant melanoma, characterized by its high metastatic potential and resistance to conventional therapies, presents a major challenge in oncology. This review explores the current status and advancements in tumor vaccines for melanoma, focusing on peptide, DNA/RNA, dendritic cell, tumor cell, and neoantigen-based vaccines. Despite promising results, significant challenges remain, including the immunosuppressive tumor microenvironment, patient heterogeneity, and the need for more effective antigen presentation. Recent strategies, such as combining vaccines with immune checkpoint inhibitors (ICIs), aim to counteract immune evasion and enhance T cell responses. Emerging approaches, including personalized neoantigen vaccines and the use of self-amplifying RNA platforms, hold promise for overcoming tumor heterogeneity and improving vaccine efficacy. Additionally, optimizing vaccine delivery systems through nanotechnology and genetic modifications is essential for increasing stability and scalability. This review highlights the potential of these innovative strategies to address current limitations, with a focus on how future research can refine and combine these approaches to improve melanoma treatment outcomes.

## 1 Introduction

Malignant melanoma, the most aggressive form of skin cancer, is responsible for 65% of skin cancer deaths, with rising global incidence and a poor prognosis in advanced stages [1,2]. Despite advances in early detection, the five-year survival rate for metastatic melanoma remains low [3]. Characterized by irregular skin lesions, melanoma is strongly linked to UV exposure [4]. Although targeted therapies like vemurafenib show initial success for BRAF V600E mutations, relapse is common, highlighting the limitations of current treatments [5]. The tumor microenvironment (TME) further hinders immune responses, complicating treatment effectiveness [6]. These challenges underscore the need for innovative therapeutic strategies, such as tumor vaccines, to enhance immune recognition of cancer cells [7]. Combining vaccines with other therapies may offer promising new solutions [6].

## 2 Immunotherapy, a Historical Background

Immunotherapy has emerged as a promising treatment strategy for various cancers, including malignant melanoma, non-small cell lung cancer (NSCLC), renal cell carcinoma, bladder carcinoma, and hepatocellular carcinoma [8,9]. Unlike traditional therapies that directly target cancer cells, immunotherapy leverages the body’s immune system to recognize and attack tumor cells [7,10]. Tumor vaccines represent a novel and potentially transformative approach in cancer immunotherapy. These vaccines aim to stimulate the immune system to recognize and eliminate cancer cells by targeting tumor-specific antigens (TSAs), a strategy that has shown promise in clinical trials [11,12].

The historical roots of cancer immunotherapy trace back to the late 19th century when German physicians Fehleisen and Busch first noted significant tumor regression following erysipelas infections [13]. This observation laid the foundation for William Bradley Coley’s pioneering use of bacterial toxins in the 1890s to stimulate the immune system against cancer [13]. Interleukin-2 (IL-2) and interferon-alpha were among the first immunotherapeutic agents approved for treating metastatic melanoma, marking an early phase in the evolution of immunotherapy for this cancer [10,14].

Recent advancements have focused on personalized neoantigen-based vaccines and mRNA vaccines. Personalized neoantigen vaccines, tailored to the unique mutations in a patient’s tumor, have shown promise in early clinical trials by inducing robust tumor-specific immune responses [15,16]. Similarly, mRNA vaccines, which gained prominence during the COVID-19 pandemic, are being explored for their potential in cancer immunotherapy due to their ability to rapidly deliver TSAs [17].

## 3 Cancer Vaccines

**Figure 1:**
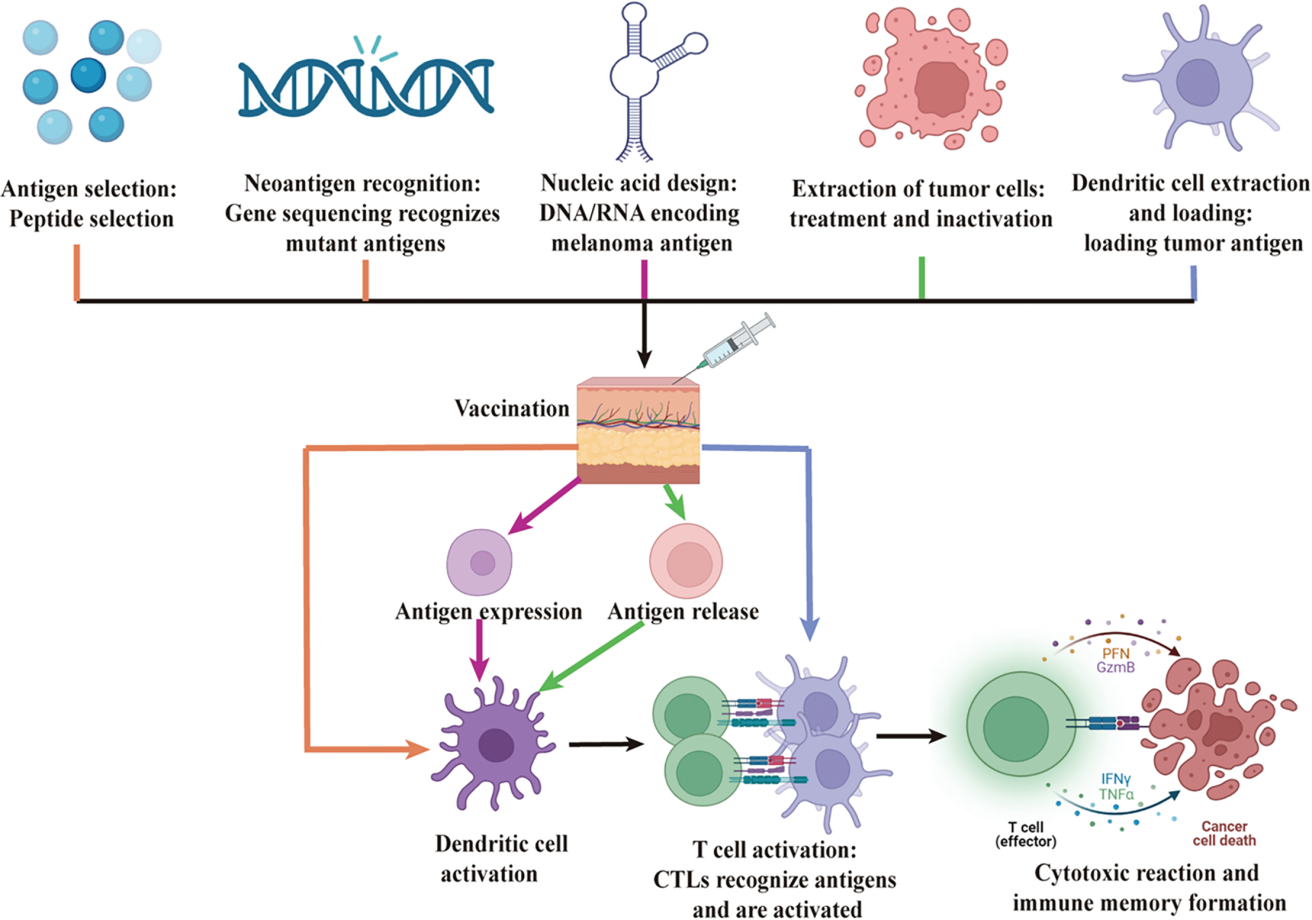
Mechanisms of action of various vaccines in melanoma immunotherapy. This figure illustrates the mechanisms of action of five different types of vaccines—Peptide Vaccines, DNA/RNA Vaccines, Dendritic Cell Vaccines, Tumor Cell Vaccines, and Neoantigen Vaccines—in the immunotherapy of melanoma. Black represents common steps. **1. Peptide Vaccines (orange):** Specific melanoma antigen peptides are selected and administered, after which they are taken up and processed by DC. This leads to the activation of CTLs, which recognize and destroy melanoma cells. **2. Neoantigen Vaccines (orange):** Neoantigens, which are unique to the patient’s tumor, are identified through genomic sequencing. A personalized vaccine is then designed and administered to induce a strong, specific T cell response aimed at targeting and eliminating melanoma cells. **3. DNA/RNA Vaccines (pink):** DNA or RNA encoding melanoma antigens is injected into the body, where host cells express the antigen. This antigen is then presented by DC, leading to the proliferation and functional activation of CTLs, ultimately resulting in a cytotoxic response against melanoma cells. **4. Tumor Cell Vaccines (green):** Inactivated whole tumor cells are used as the antigen source, stimulating the patient’s immune system. DC mediate this response, eliciting a broad T cell response targeting multiple tumor antigens. **5. Dendritic Cell Vaccines (blue):** Autologous DC are loaded with tumor antigens *ex vivo* and then reintroduced into the patient. These DC directly activate CTLs, driving a specific immune response against melanoma cells. This figure emphasizes the distinct immunological pathways and final goal of each vaccine type, which is to activate the body’s immune system, particularly CTLs, to recognize and eradicate melanoma cells. Created by BioRender

Recent advancements in cancer immunotherapy have introduced a range of vaccine strategies aimed at targeting malignant melanoma by leveraging the immune system. These include peptide vaccines derived from melanoma-associated antigens [18], DNA or RNA vaccines encoding these antigens [19,20], dendritic cell-based vaccines primed with melanoma-specific antigens [21], and whole melanoma cell vaccines that present a broad spectrum of tumor antigens [22]. Neoantigen vaccines, which are tailored to target the unique mutations in a patient’s melanoma, have emerged as a particularly promising approach in personalized medicine [15,23]. Each vaccine type engages the immune system through distinct pathways to effectively combat melanoma [24]. The various vaccine formulations and their mechanisms of action are depicted in Fig. 1.

### 3.1 Peptide Vaccines

Peptide vaccines are a promising melanoma immunotherapy strategy that use short peptides derived from tumor-associated antigens (TAAs) or TSA [25]. These vaccines aim to stimulate immune responses by presenting peptides to antigen-presenting cells (APCs), leading to the activation of cytotoxic T lymphocytes (CTLs) which target tumor cells [26]. Peptide vaccines can be divided into B cell epitope-based and T cell epitope-based vaccines, with melanoma vaccines typically focusing on T cell epitopes to activate CD8^+^ CTLs [18,27].

The mechanism involves processing peptides by dendritic cells (DC), which then present them on major histocompatibility complex (MHC) molecules. Peptides presented on MHC class I activate CD8^+^ CTLs to kill tumor cells, while MHC class II activates CD4^+^ helper T cells to enhance CTL and B cell activation [26]. The inclusion of AS02B, a combination of an oil-in-water emulsion containing monophosphoryl lipid A (MPL) and Quillaja saponaria fraction 21 (QS-21), is essential for improving the immunogenicity of the vaccine and ensuring long-term immune responses [28]. AS02B has been used in clinical trials to enhance the effectiveness of vaccines like MAGE-A3 [28].

In melanoma, several peptide vaccine candidates have been explored, including MAGE-A3, a cancer-testis antigen that is highly expressed in melanoma but not in normal tissues [29]. Clinical trials have shown that MAGE-A3 vaccines with adjuvants stimulate strong humoral and cellular immune responses, which can be further enhanced with booster doses [28]. Similarly, gp100 refers to a melanoma-associated peptide derived from the gp100 protein, a component of melanocyte differentiation antigens [30]. The gp100: 209–217(210M) peptide has been tested as a vaccine in combination with IL-2 to improve clinical outcomes in patients with advanced melanoma [30,31].

To improve peptide vaccine effectiveness, strategies such as incorporating cell-penetrating domains (CPDs) and using advanced delivery systems like water-in-oil-in-water (W/O/W) emulsions have been explored to enhance antigen presentation and CTL activation [26]. Additionally, combining peptide vaccines with immune checkpoint inhibitors (ICIs) and cytokines like IL-2 has shown promise in enhancing immune responses and improving clinical survival [18,31].

### 3.2 DNA/RNA Vaccines

DNA and RNA vaccines are innovative genetic vaccines being developed for melanoma treatment. These vaccines use the body’s own cells to produce TAAs, triggering immune responses that target and destroy cancer cells. DNA vaccines involve plasmid DNA encoding tumor antigens, while RNA vaccines utilize mRNA or circular RNA (circRNA) to deliver the same goal. RNA vaccines offer the added advantage of transient expression, reducing long-term risks [32].

The action of DNA vaccines starts with the introduction of plasmid DNA into host cells, where it is transcribed into mRNA and translated into a tumor antigen. This stimulates an immune response, activating CTLs and helper T cells to attack cancer cells [19]. RNA vaccines, on the other hand, bypass transcription by directly delivering mRNA, which is quickly translated into the antigen, offering flexibility and rapid adaptation to evolving tumor targets. Lipid nanoparticles (LNPs) improve the stability and bioavailability of mRNA vaccines [20,33].

For melanoma, the MAGE-A3 DNA vaccine targets a cancer-testis antigen found in melanoma but not normal tissues, showing promise in preclinical studies [19]. RNA vaccines like mRNA-4157 and BNT111, which target multiple tumor antigens, have shown enhanced T cell responses, especially when combined with ICIs like pembrolizumab [34,35]. Additionally, circRNA vaccines have shown superior stability and prolonged antigen expression, improving anti-tumor immunity in mouse models [36].

In summary, while DNA vaccines like MAGE-A3 face challenges in immunogenicity and delivery efficiency, RNA vaccines, including mRNA and circRNA, hold significant potential, especially when combined with ICIs. Continued research and optimization of delivery systems, such as LNPs, are crucial for enhancing the efficacy of these vaccines [37,38].

### 3.3 Dendritic Cell Vaccines

DC vaccines are a promising cancer immunotherapy, particularly for their ability to present TAAs to T cells, inducing strong anti-tumor immune responses. DC, as professional APCs, initiate immune responses by processing and presenting antigens to CTLs. The standard method for creating DC vaccines involves isolating DC from the patient, loading them with TAAs, and then reintroducing these primed DC to trigger a targeted immune response [39]. However, clinical response rates remain modest, particularly in melanoma, highlighting the need for further optimization to improve efficacy [40].

The mechanism of action for DC vaccines depends on the ability of DC to process and present tumor antigens. After loading with TAAs, DC mature and migrate to lymph nodes, where they activate CD8^+^ T cells to recognize and eliminate tumor cells [40]. Strategies like silencing programmed cell death protein 1 (PD-1) ligands using siRNA-lipid nanoparticles have been shown to enhance the immunogenicity of DC and improve antigen-specific CD8^+^ T cell responses [40]. Additionally, metabolic reprogramming of DC has emerged as a key strategy to enhance vaccine efficacy, with changes in glycolysis and oxidative phosphorylation (OXPHOS) pathways improving DC function and overcoming the immunosuppressive TME [41,42].

In melanoma, innovative strategies such as pulsing DC with iPSC-derived exosomes have shown promise in enhancing the anti-tumor immune response. This approach increases the diversity of TAAs presented to the immune system, improving the overall efficacy of melanoma immunotherapy [43]. Combining DC vaccines with ICIs like PD-1 inhibitors has also demonstrated synergistic effects, addressing the challenges posed by the immunosuppressive TME and supporting long-term anti-tumor immunity [44].

In conclusion, while DC vaccines hold significant potential for melanoma treatment, challenges such as modest clinical response rates and the need to overcome the immunosuppressive TME remain. Ongoing research focusing on genetic modulation, metabolic reprogramming, and advanced delivery systems is essential for improving DC vaccines efficacy and clinical outcomes [39,41].

### 3.4 Tumor Cell Vaccines

Tumor cell vaccines (TCV) are a promising cancer immunotherapy approach, utilizing whole tumor cells to elicit a broad and robust immune response [45]. These vaccines present a wide array of TAAs, stimulating both innate and adaptive immunity. By activating APCs like DC, TCV also engage T cells to target and destroy cancer cells. Compared to single-antigen vaccines, TCV offer greater specificity and stronger immune responses, making them valuable for treating various cancers, including melanoma [46].

TCV work by using whole tumor cells, either in their native form or genetically modified for improved immunogenicity. These tumor cells are processed by APCs, which present the antigens to T cells, initiating an immune response. For instance, incorporating immunostimulatory molecules like CpG oligodeoxynucleotides into tumor cells can enhance their ability to activate T cells and promote tumor-specific immunity [47]. In melanoma, modified B16-F10 cells expressing toll-like receptors (TLRs) and TLR7 agonists have shown promising results by inhibiting tumor growth and prolonging survival in animal models [22]. Additionally, adding molecules like VEGF164 to tumor vaccines can enhance immune responses by promoting macrophage infiltration and cancer cell death [48].

Several TCV strategies have shown potential in melanoma, including whole TCV modified with genetic alterations or irradiated to preserve antigenic properties. For example, irradiated melanoma cells combined with IL-12 have demonstrated enhanced antitumor immunity [45]. Another approach involves combining TCV with photothermal therapy and nanoparticle-loaded tumor cells, boosting immune responses at the vaccination site in melanoma models [49]. Additionally, bacteria-based autologous cancer vaccines have improved therapeutic efficacy by inducing immunogenic cell death and enhancing antigen presentation [50].

In conclusion, TCV represent a versatile and potent strategy for cancer immunotherapy, particularly in melanoma. By utilizing whole tumor cells, TCV present a comprehensive array of antigens, triggering strong and specific immune responses. Ongoing development of genetic modifications, immunostimulatory molecules, and advanced delivery techniques holds great promise for improving the clinical efficacy of TCV [49,51].

### 3.5 Neoantigen Vaccines

Neoantigen vaccines are a highly promising approach in cancer immunotherapy, leveraging tumor-specific mutations unique to each patient’s cancer. These neoantigens arise from non-synonymous mutations in tumor DNA, producing novel protein sequences absent in normal tissues, making them ideal targets for personalized cancer vaccines [15]. Advances in high-throughput sequencing and bioinformatics tools have significantly accelerated the identification of these neoantigens, allowing for vaccines tailored to the individual mutational landscape of a patient’s tumor [52].

The creation of a personalized neoantigen vaccine involves several key steps [15]. Tumor and normal tissue samples are sequenced to identify somatic mutations, and bioinformatics algorithms predict which mutations will produce peptides that can be presented on MHC molecules and recognized by T cells [53]. These predicted neoantigens are synthesized and incorporated into vaccine formulations, including synthetic long peptides (SLPs), RNA, or DC. Preclinical and early clinical studies have demonstrated that neoantigen vaccines can induce strong tumor-specific T cell responses and show preliminary evidence of antitumor activity, especially in melanoma and other cancers [23].

A key advantage of neoantigen vaccines is their high specificity, as the targeted antigens are exclusive to tumor cells, reducing the risk of autoimmunity, a concern with other cancer vaccines [54]. However, challenges remain, particularly in efficiently and safely delivering the vaccine components to elicit potent, durable immune responses [52]. Recent advances focus on improving vaccine delivery strategies, such as LNPs or viral vectors, to enhance the immunogenicity of neoantigens [52]. Despite these advancements, further research is needed to address tumor heterogeneity and the immunosuppressive TME, which can limit the effectiveness of neoantigen-based therapies [55].

In melanoma, neoantigen vaccines have shown significant promise. Clinical trials have demonstrated that these vaccines can induce strong T cell responses and improve survival rates in patients [56]. Personalized neoantigen vaccines, utilizing mRNA and dendritic cell-based platforms, are being tested to target melanoma-specific neoantigens, with promising early results [15,57,58]. Although the clinical application is still evolving, neoantigen vaccines are expected to play a critical role in transforming the treatment landscape for melanoma and other cancers [59].

In conclusion, neoantigen vaccines represent a cutting-edge, personalized approach to cancer immunotherapy, with the potential to greatly improve the precision and efficacy of cancer treatments. Ongoing research and clinical trials are refining these vaccines to enhance their efficacy and expand their application across various cancer types [60,61].

As summarized in Table 1, each type of cancer vaccine is characterized by its unique composition, advantages, and disadvantages, which collectively influence its effectiveness and clinical application in melanoma treatment.

**Table 1:** Comparison of different cancer vaccine types

Vaccine type	Advantages	Disadvantages	Composition	Application
Peptide vaccines	1. Well-defined antigens, reducing risk of autoimmunity. 2. Can be designed to target specific tumor-associated or TSAs. 3. Relatively easy to synthesize and scale for clinical use.	1. Limited by peptide stability and delivery challenges. 2. May require adjuvants (e.g., AS02B) to enhance immunogenicity, which can increase complexity. 3. Immune responses may be weaker compared to other vaccine types if not optimized.	Short peptides derived from TAAs (e.g., MAGE-A3, gp100), often used in combination with adjuvants like Freund’s adjuvant to enhance immune response.	1. Recent improvements in adjuvant systems (e.g., CpG-loaded vaccines) enhance T cell activation and improve efficacy [26]. 2. Peptide vaccines have shown efficacy when used with other therapies like IL-2, improving survival in melanoma patients [28]
DNA/RNA vaccines	1. Can encode a variety of tumor antigens, potentially addressing tumor heterogeneity. 2. mRNA vaccines have rapid production capabilities, allowing for flexible adaptation to new mutations. 3. Transient expression reduces long-term risks compared to other vaccine types.	1. Delivery and stability issues, especially for RNA vaccines. 2. DNA vaccines often face immunogenicity challenges, requiring improvements in vector design and delivery. 3. Potential for immune tolerance or insufficient immune activation without proper formulation.	Nucleic acid vaccines (DNA or mRNA) that encode TAAs or neoantigens, often delivered via LNPs.	1. mRNA vaccines have shown rapid development, including COVID-19 vaccines, with efficacy against cancers being actively studied. These vaccines generate strong T cell responses and can be used with checkpoint inhibitors for enhanced efficacy [32]. 2. DNA vaccines face challenges in immunogenicity but have potential when optimized for specific tumor types [35].
Dendritic cell vaccines	1. Can induce robust immune responses by directly activating CTLs. 2. Can present a broad range of tumor antigens, increasing chances of targeting diverse tumor variants. 3. Can be combined with ICIs for synergistic effects.	1. Clinical responses have been modest, particularly in melanoma. 2. Requires patient-specific preparation, which may limit scalability and increase costs. 3. Need for further optimization in terms of delivery methods and overcoming the TME.	DC pulsed with tumor antigens or neoantigens, sometimes genetically modified to express additional signals (e.g., PD-L1 knockdown, mRNA).	1. Provenge, the first FDA-approved DC vaccine, has shown modest efficacy in prostate cancer but has not per formed well in melanoma. 2. Other trials using personalized neoantigen-loaded DC show promise in generating specific T cell responses and boosting immunity [41].
Tumor cell vaccines	1. Can present a broad array of TAAs, leading to stronger immune responses. 2. Can stimulate both innate and adaptive immune systems, offering comprehensive immune activation. 3. Potential for use in combination with adjuvants or therapies like photothermal therapy.	1. Tumor cells must be carefully modified to ensure they are immunogenic without inducing significant side effects. 2. Risk of immune tolerance or inadequate immune responses if the tumor cells are not properly processed. 3. Preparation and scalability can be challenging due to the complexity of using whole tumor cells.	Whole tumor cells (often irradiated or otherwise modified), sometimes combined with adjuvants (e.g., CpG, IL-12) or genetically engineered to express immunostimulatory molecules.	1. Whole TCV, like those using irradiated melanoma cells or genetically modified tumor cells, show potential in activating broad immune responses but need further optimization for clinical application [22]. 2. Combining TCV with other treatments (e.g., nanoparticles, ICIs) can improve efficacy [47].
Neoantigen vaccines	1. Highly personalized, targeting specific mutations unique to a patient’s tumor. 2. High specificity for tumor cells, reducing the risk of autoimmunity. 3. Can be combined with mRNA or DC, providing flexibility in vaccine formulation.	1. Delivery can be complex, and the production process may be time-consuming and expensive. 2. Variability in tumor heterogeneity and challenges in identifying the most effective neoantigens. 3. The TME may still pose challenges to vaccine efficacy.	Custom vaccines based on tumor-specific mutations (neoantigens), which can be delivered via mRNA, peptides, or dendritic cell-based platforms.	1. Personalized neoantigen vaccines have shown high specificity, generating robust immune responses in early-phase clinical trials for mela noma and other cancers [15]. 2. Combination with checkpoint inhibitors enhances the immune response, though challenges remain in identifying all relevant neoantigens [52].

## 4 Clinical Trials

The exploration of various vaccine types in the treatment of melanoma has led to significant advancements in immunotherapy, particularly in targeting the unique challenges presented by this aggressive form of cancer. Vaccine-based therapies are designed to elicit and amplify the body’s immune response against melanoma cells, aiming to improve patient outcomes by enhancing tumor-specific immunity. The following sections provide an overview of the different vaccine types tested in clinical trials, including their mechanisms of action, clinical trial results, and potential impact on melanoma treatment.

### 4.1 Peptide Vaccines

Peptide vaccines for melanoma have been studied in several clinical trials, with varying degrees of success. These studies typically combine specific peptides with immune modulators to evaluate their therapeutic potential.

In one such trial, 24 patients with relapsed, high-risk melanoma were administered a peptide vaccine composed of melanosomal antigens such as Melan-A/MART-1, MAGE-1, gp100, and tyrosinase, along with granulocyte-macrophage colony-stimulating factor (GM-CSF). The results revealed that 20 patients showed local delayed-type hypersensitivity (DTH) reactions, indicating a strong immune response. Seven patients were able to remain relapse-free for extended periods, although 17 patients did experience disease progression, with metastases occurring in 13 during the vaccination process. Despite the challenges, the study confirmed the safety and possible clinical advantages of peptide vaccines in treating high-risk melanoma patients who have relapsed [62].

In another study, researchers tested a combination of peptide vaccines, GM-CSF, and low-dose IL-2 in patients with melanoma at stages II, III, or IV who had undergone resection. The peptides used were MART-1a, gp100(207–217), and survivin. While the combination stimulated immune responses, it was found that adding IL-2 did not improve the results. Furthermore, IL-2 administration increased the levels of T regulatory cells (Tregs), which are known to suppress immune activity, thus potentially reducing the effectiveness of the vaccine [63].

A comprehensive review of multiple clinical trials examining peptide vaccines for melanoma demonstrated that the addition of IL-2 to peptide vaccines could enhance disease control in patients, as compared to IL-2 alone. Moreover, patients who exhibited a tumor-specific immune response showed improved survival rates. However, the review emphasized that further studies are necessary to conclusively determine whether peptide vaccines offer greater clinical benefits than other treatment modalities [64].

### 4.2 DNA/RNA Vaccines

Recent clinical trials evaluating DNA and RNA vaccines for melanoma have shown promising results, demonstrating their potential to provoke strong immune responses in patients.

In one Phase I/II study, a GM-CSF DNA vaccine was used as an adjuvant in a multi-peptide cancer vaccine for patients with advanced melanoma. The trial included 19 patients and focused on assessing the safety and immune-stimulating effects of the vaccine. The findings indicated that the vaccine was generally well tolerated, with only mild injection-site reactions being reported. Notably, 42% of the patients developed CD8^+^ T-cell responses, suggesting that the vaccine was effective in enhancing anti-tumor immunity [65].

Another study explored the use of a DNA vaccine encoding tyrosinase epitopes delivered intranodally in Stage IV melanoma patients. The vaccine was injected directly into a groin lymph node, targeting local APCs. The treatment showed minimal toxicity, and immune responses to tyrosinase were detected in 11 out of 26 patients. Despite the lack of clinical responses, the survival rate was surprisingly high, with 16 of the 26 patients alive after a median follow-up of 12 months [66].

RNA vaccines have also demonstrated significant potential. One study involving an mRNA vaccine encoding TAAs induced both humoral and cellular immune responses. The use of LNPs as a delivery system was shown to improve the stability and effectiveness of the mRNA vaccine, reinforcing the versatility of RNA-based platforms for cancer immunotherapy [67]. These promising results underline the growing interest and ongoing development of DNA and RNA vaccines as effective melanoma treatments.

### 4.3 Dendritic Cell Vaccines

Clinical trials have shown that DC vaccines have the potential to induce strong immune responses and improve clinical outcomes in melanoma treatment.

In a study by Bedrosian, the feasibility, safety, and immunogenicity of peptide-pulsed mature DC vaccines were evaluated in 27 patients with metastatic melanoma. The study found that intranodal (IN) injection significantly enhanced CD8^+^ T-cell responses and DTH reactions compared to intravenous (IV) and intradermal (ID) injections. Therefore, IN injection was recommended as the preferred route for DC vaccine administration [68].

A randomized phase II clinical trial by Dillman compared autologous DC vaccines with autologous TCV in 42 patients with metastatic melanoma. The results showed that the median survival of patients receiving DC vaccines was 43.4 months, compared to 20.5 months for those receiving TCV. DC vaccines significantly prolonged patient survival with relatively mild side effects [69].

In addition, a phase II trial by Geskin investigated three different antigen-loading methods in DC vaccines for 15 patients with metastatic melanoma. Although the effectiveness of DC vaccines alone was limited, the study suggested that combining DC vaccines with other immunotherapies, such as checkpoint inhibitors, might enhance their therapeutic potential [70].

### 4.4 Tumor Cell Vaccines

TCV, which use whole tumor cells to stimulate an immune response, have demonstrated varying success levels in clinical trials.

In a randomized phase II trial by Dillman, autologous TCV were compared with dendritic cell vaccines (DCV) in metastatic melanoma patients. The study showed that DCV was associated with significantly longer survival, with a median survival of 43.4 months compared to 20.5 months for TCV. The results suggested that DCV might offer superior clinical outcomes due to its more efficient presentation of TAAs [69].

Leong’s study evaluated the combination of recombinant human granulocyte-macrophage colony-stimulating factor (rhGM-CSF) with autologous melanoma vaccines in stage IV melanoma patients. In this trial of 20 patients, 10% achieved a complete response, and another 10% had partial responses. The addition of rhGM-CSF appeared to enhance the immune response, leading to tumor regression in some patients, suggesting its potential as an adjuvant in TCV [71].

In a study by Lotem, the use of an autologous melanoma cell vaccine as adjuvant therapy was explored in high-risk melanoma patients. The trial included 43 patients, and the results showed that patients who developed a strong DTH response to the vaccine had significantly better disease-free and overall survival (OS) rates. These findings suggest that the intensity of the immune response correlates with improved clinical outcomes, supporting the use of TCV in adjuvant melanoma therapy [72].

### 4.5 Neoantigen Vaccines

Neoantigen vaccines have shown significant promise in melanoma treatment by targeting tumor-specific mutations unique to each patient. Personalized neoantigen vaccines, developed through advances in sequencing and bioinformatics, have demonstrated strong tumor-specific immunogenicity and preliminary antitumor activity.

A notable clinical trial tested the personalized peptide-based neoantigen vaccine EVX-01, combined with the novel CAF®09b adjuvant, in patients with metastatic melanoma. This study showed that the vaccine induced long-lasting T-cell responses specific to neoantigens, with all patients demonstrating immune responses. Importantly, the vaccine was well-tolerated with no severe adverse events, supporting its safety and promising efficacy in melanoma treatment [73].

Additionally, a significant study by Hu investigated the long-term effects of the NeoVax vaccine, which targets multiple personalized neoantigens. The results revealed persistent neoantigen-specific T-cell responses and epitope spreading, suggesting durable immune memory and effective tumor cell killing. Six out of eight patients remained disease-free at a median follow-up of nearly four years, underscoring the vaccine’s potential for long-term cancer control [57].

### 4.6 Combination Therapies

Combination therapies in melanoma have gained significant attention due to their potential to enhance immune responses and improve patient outcomes. Tumor vaccines, when combined with ICIs, have demonstrated synergistic effects that bolster the body’s ability to combat cancer. One study emphasized the potential of combining vaccines and oncolytic viral therapies with ICIs, noting that such combinations can convert ‘cold’ tumors into ‘hot’ tumors, enhancing their vulnerability to immune attacks and improving survival rates in metastatic melanoma [74].

Other research has examined the pairing of vaccines with ICIs, such as sipuleucel-T and talimogene laherparepvec (T-VEC), which have shown promise in eliciting robust antitumor responses. These vaccines help prime the immune system, expand immune responses, and enhance immune function within the TME [75]. Moreover, the combination of BRAF inhibitors and MEK inhibitors with ICIs for BRAF-mutant melanomas has proven effective. Anti-PD-1 agents, like nivolumab and pembrolizumab, are central to these combination strategies, offering high efficacy and low toxicity [76].

In another study, a novel injectable polypeptide hydrogel was developed to co-deliver a tumor vaccine alongside dual ICIs. This method not only increased the number of activated effector CD8^+^ T cells but also reduced the ratio of Tregs, thereby strengthening overall antitumor immunity [77].

Recent advancements in melanoma treatment, particularly in neoadjuvant therapy, are crucial for melanoma vaccine development. Neoadjuvant immunotherapy with nivolumab and ipilimumab has shown significant promise, improving event-free survival compared to adjuvant therapy. For example, a study by Georgina Long et al. demonstrated that neoadjuvant therapy for resectable stage III melanoma resulted in an estimated 12-month event-free survival of 83.7%, compared to 57.2% with adjuvant therapy. Additionally, 59% of patients had a major pathological response, emphasizing the potential of neoadjuvant approaches to improve outcomes [78].

Integrating melanoma vaccines with neoadjuvant immunotherapies could further enhance immune responses prior to surgery, increasing tumor vulnerability to immune attacks. This combination could significantly improve treatment efficacy, survival rates, and reduce recurrence in high-risk melanoma patients.

Tables 2–6 summarizes the clinical trials of various vaccines against melanoma, highlighting the vaccine types, study designs, patient cohorts, adjuvants used, stages of melanoma targeted, and the observed outcomes. This table serves as a comprehensive reference to understand the effectiveness and safety profiles of these vaccines, supporting the ongoing development and optimization of melanoma immunotherapy strategies.

**Table 2:** Clinical trials of peptide vaccines

Vaccine type	Study design	Number of patients	Adjuvant/ Treatment	Stage of melanoma	Outcomes	Clinical Trials.gov identifier	Reference
Peptide vaccine	Phase II	24	GM-CSF plus antigenic peptide	Locally Advanced Melanoma	Improved immune response in some patients but limited clinical efficacy	——	[62]
Peptide vaccine	Phase I	19	GM-CSF, IL-2	Resected Stage II–IV	CTL responses observed in most patients, but neither increased GM-CSF dose nor IL-2 improved immune response significantly	——	[63]

**Table 3:** Clinical trials of DNA/RNA vaccines

Vaccine type	Study design	Number of patients	Adjuvant/Treatment	Stage of melanoma	Outcomes	Clinical Trials. gov identifier	Reference
DNA vaccine	Phase I/II	19	GM-CSF DNA as adjuvant for multipeptide vaccine	Advanced Melanoma	Safe with some immune response; ongoing assessment of efficacy	–	[65]
DNA vaccine	Phase I	26	Intranodal delivery of plasmid DNA	Stage IV	Safe with some T-cell responses, but limited overall efficacy	–	[66]
DNA vaccine	Phase I	18	Tyrosinase DNA vaccine	Stage III/IV	Safe with CD8^+^ T-cell responses observed in some patients	–	[67]

**Table 4:** Clinical trials of dendritic cell vaccines

Vaccine type	Study design	Number of patients	Adjuvant/Treatment	Stage of melanoma	Outcomes	Clinical Trials.gov identifier	Reference
Peptide-pulsed dendritic cell vaccine	Phase I, randomized	27	Peptide-pulsed mature dendritic cells administered via intranodal (IN), intravenous (IV), or intradermal (ID) routes	Metastatic Melanoma	Safe with some immune response; ongoing assessment of efficacy	–	[68]
Autologous dendritic cell vaccine vs. Autologous tumor cell vaccine	Randomized Phase II, 5-year follow-up	42 (dendritic cell) vs. 54 (tumor cell)	Dendritic cells loaded with autologous tumor antigens vs. autologous tumor cell vaccines	Metastatic Melanoma	Safe with some T-cell responses, but limited overall efficacy	NCT00948480	[69]
Dendritic cell vaccine (Three Antigen-Loading Methods)	Comparative Phase I/II	15	Dendritic cells loaded with antigens via three different methods: mRNA electroporation, peptide pulsing, and direct antigen loading	Metastatic Melanoma	Peptide-pulsed and mRNA electroporated dendritic cells showed better immunogenicity compared to direct antigen loading; no significant difference in clinical outcomes observed	–	[70]

**Table 5:** Clinical trials of tumor cell vaccines

Vaccine type	Study design	Number of patients	Adjuvant/Treatment	Stage of melanoma	Outcomes	Clinical Trials.gov identifier	Reference
Autologous melanoma vaccine	Phase I/II	20	Recombinant human granulocyte macrophage-colony stimulating factor (rhGM-CSF) combined with autologous melanoma vaccine	Metastatic melanoma	Tumor regression observed in some patients; safe with manageable toxicity	–	[71]
Autologous cell vaccine	Phase II	43	Post-operative autologous cell vaccine	AJCC Stages III and IV	Improved disease-free survival (DFS) and overall survival (OS) in patients, particularly those with a positive delayed-type hypersensitivity (DTH) response	–	[72]

**Table 6:** Clinical trials of neoantigen vaccines

Vaccine type	Study design	Number of patients	Adjuvant/Treatment	Stage of melanoma	Outcomes	Clinical Trials.gov identifier	Reference
Peptide-based neoantigen vaccine	Personalized Therapy, Phase I/II	5	Neoantigen vaccine (EVX-01) with CAF®09b adjuvant	Metastatic Melanoma	Induced strong CD8^+^ T-cell responses; ongoing studies to assess long-term clinical efficacy	NCT03715985	[73]
Personalized neoantigen vaccine	Phase I	8	Personalized vaccine targeting patient-specific neoantigens	Advanced Melanoma	Induced persistent memory T cell responses and epitope spreading; enhanced long-term immunity	NCT01970358	[57]

## 5 Future Directions

Future cancer vaccine research should focus on enhancing tumor antigen immunogenicity, particularly in melanoma. Combining vaccines with ICIs, such as anti-PD-1/PD-L1(Programmed death-ligand 1) and anti-CTLA-4, can counteract immune evasion and boost T cell responses in melanoma patients [79,80]. Targeting the cyclic adenosine monophosphate (cAMP) pathway in the TME holds promise for reducing immunosuppression and improving vaccine effectiveness in melanoma [81].

Advances in personalized melanoma vaccines, particularly neoantigen-based approaches, are key to addressing tumor heterogeneity in melanoma. Enhanced sequencing and bioinformatics can speed up antigen identification, lowering the cost and time required for personalized vaccine development [82]. Multi-antigen strategies, such as targeting multiple melanoma-specific antigens, also show potential in ensuring broader, longer-lasting immune responses [83].

For vaccine delivery, optimizing nanotechnology-based systems like LNPs and hydrogels is critical for stability and scalability in melanoma vaccines [84]. Innovations such as self-amplifying RNA (saRNA) and circRNA vaccines can improve antigen expression and immune activation, while minimizing safety concerns [85]. Combining these platforms with ICIs or other adjuvants could enhance therapeutic outcomes in melanoma patients [86].

Genetically modified vaccines, which enhance antigen presentation or include immune-boosting components, have significant potential in melanoma treatment [82]. Research into modulating the TME, overcoming immunosuppressive factors like Tregs and MDSCs, and enhancing effector T cell recruitment is crucial for further improvement [87].

Collaboration between researchers, clinicians, and regulatory bodies will be essential for translating these advances into clinical practice, making melanoma vaccines safer, more effective, and accessible [88].

## 6 Conclusion

In conclusion, tumor vaccines hold immense potential as a therapeutic strategy for melanoma, leveraging the body’s immune system to target and destroy cancer cells. The advances in personalized medicine, particularly with neoantigen vaccines, offer a promising avenue for individualized treatment plans that could improve patient outcomes. However, despite these advancements, several challenges and limitations must be addressed to optimize the efficacy of tumor vaccines.

## Data Availability

Not applicable.

## Abbreviations

APCs: Antigen-presenting cells
AS02B: Adjuvant System 02B
BRAF: B-Raf proto-oncogene
CAR-T: Chimeric antigen receptor T cells
circRNA: Circular RNA
CTLA-4: Cytotoxic T-lymphocyte-associated protein 4
CTLs: Cytotoxic T lymphocytes
DC: Dendritic cell
DCV: Dendritic cell vaccines
DFS: Disease-free survival
DTH: Delayed-type hypersensitivity
GM-CSF: Granulocyte-macrophage colony-stimulating factor
gp100: Glycoprotein 100
HLA: Human leukocyte antigen
ICIs: Immune checkpoint inhibitors
ID: Intradermal
IL-2: Interleukin-2
IL-12: Interleukin-12
IN: Intranodal
iPSC: Induced pluripotent stem cell
IV: Intravenous
LNPs: Lipid nanoparticles
MAGE-A3: Melanoma-associated antigen 3
MDSCs: Myeloid-derived suppressor cells
MEK: Mitogen-activated protein kinase kinase
MHC: Major histocompatibility complex
mRNA: Messenger RNA
MPL: Monophosphoryl lipid A
NeoVax: Personalized neoantigen vaccine
NSCLC: Non-small cell lung cancer
OS: Overall survival
OXPHOS: Oxidative phosphorylation
PD-1: Programmed cell death protein 1
PD-L1: Programmed death-ligand 1
QS-21: Quillaja saponaria fraction 21
rhGM-CSF: Recombinant human granulocyte-macrophage colony-stimulating factor
saRNA: Self-amplifying RNA
siRNA: Small interfering RNA
SLPs: Synthetic long peptides
TAA: Tumor-associated antigen
TCV: Tumor cell vaccines
TLR: Toll-like receptor
TME: Tumor microenvironment
Tregs: Regulatory T cells
TSA: Tumor-specific antigen
T-VEC: Talimogene laherparepvec
VEGF: Vascular endothelial growth factor
